# Virtual Oncology Appointments during the Initial Wave of the COVID-19 Pandemic: An International Survey of Patient Perspectives

**DOI:** 10.3390/curroncol28010065

**Published:** 2021-01-22

**Authors:** Jonathan M. Loree, Hallie Dau, Nevena Rebić, Alyssa Howren, Louise Gastonguay, Helen McTaggart-Cowan, Sharlene Gill, Kanwal Raghav, Mary A. De Vera

**Affiliations:** 1 BC Cancer, Vancouver, BC V5Z 4E6, Canada; Jonathan.Loree@bccancer.bc.ca (J.M.L.); hcowan@bccrc.ca (H.M.-C.); sgill@bccancer.bc.ca (S.G.); 2 Department of Medicine, Division of Medical Oncology, Faculty of Medicine, University of British Columbia, Vancouver, BC V5Z 4E6, Canada; 3 Faculty of Pharmaceutical Sciences, University of British Columbia, 2405 Wesbrook Mall, Vancouver, BC V6T 1Z3, Canada; hallie.dau@ubc.ca (H.D.); nevena.rebic@ubc.ca (N.R.); alyssa.howren@ubc.ca (A.H.); louisega@mail.ubc.ca (L.G.); 4 Collaboration for Outcomes Research and Evaluation, Vancouver, BC V6T 1Z3, Canada; 5 Faculty of Health Sciences, Simon Fraser University, Burnaby, BC V5A 1S6, Canada; 6 Department of GI Medical Oncology, The University of Texas MD Anderson Cancer Center, 1515 Holcombe Blvd, Houston, TX 77030, USA; KPRaghav@mdanderson.org

**Keywords:** cancer, COVID-19, telemedicine, telehealth, virtual health

## Abstract

There has been rapid implementation of virtual oncology appointments in response to the COVID-19 pandemic, particularly in its first wave. Our objective was to assess patterns and perspectives towards virtual oncology appointments during the pandemic among patients with cancer undergoing active treatment. We conducted an international Internet-based cross-sectional survey. Participants were eligible if they (**1**) were ≥18 years of age; (**2**) had been diagnosed with cancer (**3**) were currently undergoing cancer treatment, and (**4**) spoke English or French. Between 23 April 2020 and 9 June 2020, 381 individuals accessed the survey, with 212 actively undergoing treatment for cancer, including 27% with colorectal, 21% with breast, 7% with prostate and 7% with lung cancer. A total of 52% of respondents were from Canada and 35% were from the United States. Many participants (129, 62%) indicated having had a virtual oncology appointment during the COVID-19 pandemic and most were satisfied with their experience (83%). We found older participants (≥50 years; adjusted OR 0.22, 95% CI 0.06 to 0.85 compared to <50 years) and those with shortest duration of treatment (≤3 months; adjusted OR 0.06; 95% CI 0 to 0.69 compared to >12 months) were less likely to be satisfied with virtual oncology appointments. Virtual health platforms used differed across countries with higher telephone use in Canada (87%) and other countries (86%) as compared to the United States (54%; *p*-value < 0.05), where there was higher use of video conferencing. Altogether, our findings demonstrate favorable patient perspectives towards virtual oncology appointments experienced during the first wave of the COVID-19 pandemic.

## 1. Introduction

Delivering care to patients with cancer during the COVID-19 pandemic is particularly challenging due to the competing risks of morbidity and mortality from cancer and COVID-19 infection in this vulnerable population [1]. As a result of the COVID-19 pandemic, particularly during the first wave, healthcare systems worldwide shifted their model of care to utilize virtual approaches, wherever possible [2]. It is important to learn from patient experiences with this care delivery to inform best practices and optimize care as the COVID-19 pandemic continues and beyond. Prior to the pandemic, the potential benefits and promise of telehealth and telehealth interventions among patients with cancer had been reported [3,4]. We aimed to assess the perspectives of patients with cancer towards virtual oncology appointments during the first wave of the COVID-19 pandemic.

## 2. Methods

### 2.1. Study Design and Participants

This study is nested within an international Internet-based cross-sectional study aimed to better understand the impacts of COVID-19 on the care and outcomes of patients with cancer. Participants were eligible if they (1) were ≥18 years of age; (2) were currently undergoing cancer treatment, and (3) spoke English or French. We recruited participants both online using the authors’ social media channels as well as those of cancer organizations that supported our efforts.

### 2.2. Survey

Participants completed an online survey which was designed with input from patient research partners and clinicians. The survey comprised of 85 questions in nine sections. Relevant sections for this current study include: demographic information, cancer characteristics, current cancer treatment(s), experiences and satisfaction with virtual oncology appointments during the COVID-19 pandemic, and use of technology for healthcare (based on the National Cancer of Institute’s Health Information National Trends Survey on individuals’ use of cancer-related information [5]).

### 2.3. Analysis

We conducted cross-sectional analyses on non-missing survey responses, using descriptive statistics and chi-square tests for comparisons. We created a binary categorical outcome representing participants’ satisfaction with virtual oncology appointments and evaluated determinants using multivariable logistic regression models, among those who indicated having such visits during the first wave of the COVID-19 pandemic. Potential determinants included sociodemographic and cancer characteristics as well as appointment characteristics (e.g., platforms used). Analyses were conducted using SAS 9.4. Ethics were approved by the University of British Columbia.

## 3. Results

Corresponding to the first wave of the COVID-19 pandemic, between 23 April 2020–9 June 2020, 381 individuals accessed the survey with 212 actively undergoing treatment for cancer and comprising our study sample (Table 1). The most represented cancer types were colorectal (27%), breast (21%), prostate (7%), and lung (7%). The majority of participants were female (142, 67%) and diagnosed with stage IV cancer (97, 46%). When asked about changes to specific aspects of oncology care during the COVID-19 pandemic, we found that these were largely initiated by the healthcare provider, particularly with respect to delays in chemotherapy (92%) and radiation (92%). We also noted a substantial proportion of cancelled or delayed clinical trials that were initiated by healthcare provider(s) (83%).

**Figure 1:**
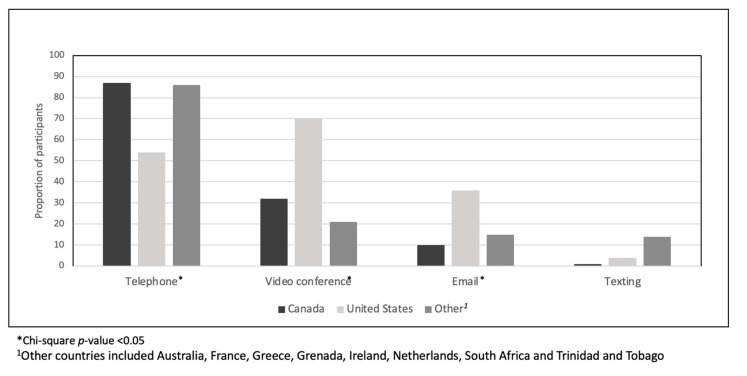
Country-wise comparison of platforms used for virtual oncology appointment(s) during the COVID-19 pandemic.

With respect to patterns of virtual oncology appointments during the first wave of the COVID-19 pandemic, many participants (129, 62%) indicated having a virtual oncology appointment. While the majority (164, 79%) of respondents reported not having the option to choose between a virtual or in-person appointment, 125/164 (76%) indicated that this did not bother them. We asked participants to indicate all of the platform(s) they have used for virtual oncology appointments (Figure 1) and we noted differences between countries including higher telephone utilization in Canada (87%) and other countries (86%), as compared to the United States (US) (54%; chi-square *p*-value < 0.05). Conversely, we found a higher use of video conferencing in the US (70%) as compared to Canada (32%) and other countries (21%; chi-square *p*-value < 0.05). Though less frequent, we found that other countries had the highest use of email (36%).

Among participants that had virtual oncology appointments during the first wave of the COVID-19 pandemic, most participants (106, 83%) indicated being satisfied with their virtual oncology appointments. Determinants of satisfaction with virtual oncology appointments included current age, with those aged ≥50 years 78% less likely to be satisfied (adjusted odds ratio (OR), 0.22; 95% confidence interval (CI), 0.66 to 1.07) compared to participants <50, and cancer treatment duration, with those who have been in treatment for ≤3 months being 94% less likely to be satisfied (adjusted OR 0.06; 95% CI, 0.00 to 0.60), compared to those who had been experiencing treatment for >12 months (Table 2).

When querying further experiences and perspectives, among 72 respondents who participated in virtual oncology appointments that did not use video conferencing, 36% indicated they preferred to see their healthcare provider visually through video, 49% were indifferent, and 15% preferred not to see their provider on video. As regards receiving difficult news, most participants ranked appointments in-person (85, 72%) and telephone (19, 16%) above video conferencing (14, 12%). Finally, most participants (155, 75%) shared that they felt supported by their cancer care provider during the first wave of the COVID-19 pandemic.

In terms of access to and use of technology for healthcare, most participants indicated having a smartphone, followed by a laptop, then tablet (Table 1). Though the majority of participants owned more than one device (171, 85%), we also noted a few (31, 15%) that owned a single device. When asked about utilization in the past 12 months, the majority of participants indicated that they have used technology to look for medical information (93%) and look up results from a medical test (77%). As well, 66% of respondents indicated that they have used technology for a virtual cancer appointment by telephone and 44% by video conference in the past 12 months.

## 4. Discussion

The COVID-19 pandemic has led to a rapid shift towards the virtual delivery of oncology care, particularly with respect to appointments that do not typically require physical care, such as treatment follow-up. In assessing care patterns and patient perspectives, we found that nearly two-thirds of study participants reported having a virtual oncology appointment during the first wave of the COVID-19 pandemic. This corroborates findings of a recent survey of Canadian medical oncologists that reported 82% used some form of virtual care [6]. Furthermore, our finding that the majority of patients (83%) were satisfied with their virtual oncology appointments provides reassurance to healthcare providers.

Identified patient experiences and perspectives on virtual oncology appointments during the first wave of the COVID-19 pandemic have implications in terms of recommendations for ensuring patient-centered care to individuals undergoing cancer treatment as the pandemic progresses. Our results identify patient subgroups that could be provided additional support. For example, older patients (≥50 years) and those who have recently started their cancer treatment (≤3 months) were less likely to be satisfied with virtual oncology appointments. As well, since the majority of participants did not have a preference towards visibly seeing their healthcare provider during virtual appointments, resources could prioritize telephone-based delivery, which can be simpler. Virtual appointments require distribution of a secure link and ensuring the provider is ready on time to activate a meeting, while telephones require less clerical support.

We also surveyed health seeking behaviors in terms of the use of technologies for healthcare as well as queried care patterns. Although the use of digital technologies was previously assessed by Abrol et al. among adolescents and young adults with cancer in 2017 [7] and more recently among individuals with young-onset and average-age onset colorectal cancer [8], to our knowledge this has not been assessed across a wider range of cancer patient populations. Such assessments are needed in order to align patients’ digital literacy, that is, “the ability to search, access, and understand health information from electronic sources [9]” with virtual healthcare delivery.

We found jurisdictional differences in platforms used, notably, a higher utilization of telephone appointments in Canada and other countries for virtual oncology appointments, as compared to the US where higher use of video conferencing was reported by participants. These differences may be explained by reimbursement models. In Canada, most medical oncologists are salaried and there are no incentives to one form of care over another. However, in the United States with a largely fee-for-service model, there are financial incentives to using interactive video technology which carries a higher billing code than telephone calls. Although Medicare/Medicaid modified billing for telehealth visits during the COVID-19 pandemic to be remunerated like in-person visits in the US [10], the infrastructure and care pathways to support video over telephone visits due to compensation differences may still exist. In Canada, a 2019 discussion paper highlighting issues and challenges of virtual healthcare delivery by the Canadian Medical Association [11], led to the establishment of a Virtual Care Task Force [12]. With the COVID-19 pandemic necessitating the rapid implementation of virtual healthcare delivery—particularly with vulnerable patient populations such as those with cancer—lessons can be taken to inform continued efforts towards the development and uptake of virtual healthcare.

Limitations of our study warrant discussion. Our study is limited by sample size and is largely based on residents of high-income countries with a post-secondary education, with a bias towards individuals with Internet access. While these participant characteristics may explain their favorable perspectives towards virtual healthcare delivery, this also highlights the importance of gathering perspectives of under-represented sub-populations of cancer patients. Our sample also predominantly included those with advanced cancer stage, which may be due to the fact that these patients experience active treatment for longer periods of time. While our survey comprised a range of questions regarding experiences during the COVID-19 pandemic, when considering potential participant burden, we may have missed specific issues that may be relevant to this study (e.g., having a landline telephone, number of virtual oncology appointments attended).

Our findings demonstrate favorable patient perspectives towards virtual oncology appointments during the first wave of the COVID-19 pandemic and suggest this may be a viable approach for introducing greater efficiency into oncology care beyond the COVID-19 pandemic.

## Figures and Tables

**Table 1:** Survey responses.

**Demographic Characteristics**	
Age, year (median (range))	54 (21, 93)
Age	
<50 years	84 (40)
≥50 years	128 (60)
Female, n (%)	142 (67)
Country, n (%)	
Canada	111 (52)
United States	74 (35)
Other ^a^	27 (13)
Highest level of education completed, n (%)	
Secondary (elementary, high school)	41 (19)
Post-secondary (university, college, technical school)	171 (81)
Tested for COVID-19, n (%)	
No	165 (84)
Yes	32 (16)
Negative test	26 (81)
Positive test	2 (6)
Waiting for results	3 (9)
Prefer not to answer	1 (3)
**Cancer Characteristics**	
Cancer type ^b,c^, n (%)	
Colorectal	58 (27)
Breast	45 (21)
Prostate	15 (7)
Lung	14 (7)
Other	90 (42)
Cancer stage, n (%)	
0	2 (1)
I	21 (10)
II	23 (11)
III	36 (17)
IV	97 (46)
Do not know	30 (14)
Cancer treatment duration, n (%)	
≤3 months	41 (20)
3 to 12 months	58 (28)
>12 months	109 (52)
Number of treatment modalities, n (%)	
Single	104 (49)
Multiple	106 (50)
None	2 (1)
Type of treatment ^c^, n (%)	
Infusion chemotherapy	105 (50)
Surgery	68 (32)
Radiation	53 (25)
Oral chemotherapy	47 (22)
Immunotherapy	33 (16)
Other	65 (31)
Use of Technology for Healthcare	
Digital devices owned ^c^, n (%)	
Smartphone	184 (87%)
Laptop	141 (67%)
Tablet	131 (62%)
Desktop computer	82 (39%)
Basic cell phone	17 (8%)
Health-related use of digital device ^d^, n (%)	
Look for medical information	190 (93%)
Look up results from a medical test	161 (77%)
Participate in a virtual medical appointment over video conference	89 (44%)
Participate in a virtual medical appointment over telephone	135 (66%)

^a^ Other countries included Australia, France, Greece, Grenada, Ireland, Netherlands, South Africa and Trinidad and Tobago; ^b^ Most frequent cancers among respondents reported; ^c^ Not discrete as participants can report ≥1 type (e.g., proportions do not add to 100%); ^d^ In the past 12 months.

**Table 2:** Multivariable logistic regression of predictors of satisfaction with virtual oncology appointments.

	Odds Ratio	95% Confidence Interval
Years from cancer diagnosis	0.84	(0.66, 1.07)
Age		
<50 years (ref)		
≥50 years	0.22	(0.06, 0.85)
Location		
Other (ref)		
Canada	2.67	(0.63, 11.38)
United States	2.79	(0.32, 24.33)
Gender		
Male (ref)		
Female	0.33	(0.08, 1.28)
Cancer treatment length		
>12 months (ref)		
3 to 12 months	1.04	(0.26, 4.08)
≤3 months	0.06	(0.00, 0.69)
Platform used for virtual oncology appointment ^a^		
No video conference (ref)		
Video conference	0.97	(0.21, 4.43)
No telephone (ref)		
Telephone	1.00	(0.19, 5.31)
No texting (ref)		
Texting	0.74	(0.02, 22.84)
No email (ref)		
Email	0.42	(0.04, 4.67)
Treatment modality ^a^		
No radiation (ref)		
Radiation	0.75	(0.18, 3.09)
No oral chemotherapy (ref)		
Oral chemotherapy	0.88	(0.19, 4.14)
No infusion chemotherapy (ref)		
Infusion chemotherapy	0.70	(0.13, 3.74)
No surgery (ref)		
Surgery	1.07	(0.31, 3.7)
No immunotherapy (ref)		
Immunotherapy	1.58	(0.37, 6.82)
No other (ref)		
Other	0.38	(0.04, 3.49)

^a^ Variables are not discrete.
